# The Infraorbital Foramen in a Sample of the Lebanese Population: A Radiographic Study

**DOI:** 10.7759/cureus.6381

**Published:** 2019-12-14

**Authors:** Sayde Sokhn, Ronald Challita, Anthony Challita, Raymond Challita

**Affiliations:** 1 Oral and Maxillofacial Imaging, Lebanese University, Beirut, LBN; 2 Obstetrics and Gynecology, Faculty of Medicine, Lebanese University, Beirut, LBN; 3 Periodontology and Implantology, Lebanese University, Beirut, LBN; 4 Plastic and Reconstructive Surgery, Faculty of Medicine, Lebanese University, Beirut, LBN

**Keywords:** nerve block, infraorbital foramen, cbct, accessory infra orbital foramen, ethnic variations

## Abstract

Purpose

The infraorbital foramen (IOF) is an important structure in the maxillofacial region through which important structures pass. Wide variability in the shape and location of the infraorbital foramen among different populations and ethnic groups is present. So we conducted this study to specify the IOF shape, the presence of accessory foramina, and the IOF location with respect to anatomic landmarks in the Lebanese population.

Patients and method

A cross-sectional retrospective study was conducted on cone-beam computed tomography (CBCT) scans of 105 Lebanese adult patients. Images were reviewed and the shape, diameter, and location of the IOF were recorded. The presence of an accessory foramen was also noted. Then, SPSS version 21 (IBM Corp., Armonk, NY, US) was used for the statistical analysis.

Results

Concerning the distances from the IOF to the anatomic landmarks, the distance from the IOF to the infraorbital margin measured 7.98 ± 1.41 mm, to the lateral nasal wall 10.61 ± 2.39 mm, and to the midline 24.71 ± 2.09 mm. When distances were compared, a statistical difference was only identified in the distance between the IOF and the lateral nasal wall (p=0.00), and the distance between the IOF and the middle of the face (p=0.016) between genders. For the shape of the IOF, 54.8% of the IOF were circular in shape, and this shape was the most common shape in females. An accessory foramen was present in 8.6% of the cases. Finally, the mean diameter of the foramina measured 3.71 ± 0.63 mm.

Conclusion

The IOF shows a lot of variability between different populations. Thus, the exact location should always be remembered during an infraorbital nerve (ION) block, during maxillofacial surgeries, and during esthetic procedures involving the facial region in order to prevent unnecessary complications.

## Introduction

The infraorbital foramen (IOF) is present on the maxillary bone. The infraorbital nerve, vein, and artery pass through this foramen [1].

The infraorbital nerve is an essential facial sensory nerve. It originates from the trigeminal ganglion, passes through the facial skeleton, and exits through the infraorbital foramen [1]. It is responsible for sensation in the upper cheek, the inferior eyelid, part of the nose, the upper lip, the maxillary sinus, and some of the maxillary teeth [2].

The exact anatomic location of the infraorbital nerve is crucial in various procedures. An infraorbital nerve (ION) block is a procedure performed in anesthesia during maxillofacial surgeries, in the management of postoperative pain, and in the treatment of trigeminal neuralgia [3-5]. Furthermore, this nerve is also important in various surgeries, such as rhinoplasty, facial tumor surgeries, orbital floor fractures, Le Fort I fractures, malar fractures, and malar and facial implant placement [6-7]. It is worth noting that the presence of an accessory infraorbital foramen adds to the complexity of this area and should be remembered by anesthesiologists and maxillofacial surgeons [8].

Similarly, the exact anatomic location of the infraorbital artery is critical in various procedures, especially in plastic surgery where flaps based on the infraorbital artery can be used for nasal ala reconstruction [9]. The location of this artery is also present in one of the facial danger zones, and a dermal fillers injection in this area can lead to arterial occlusion with associated stroke and blindness [10].

Multiple studies in the literature have shown wide variability in the shape and location of the infraorbital foramen among different populations and ethnic groups, which could be problematic for many surgeons [11].

Despite its clinical significance, no data is present on the IOF shape and location in the Lebanese population. Hence, the present study was conducted to specify the IOF shape, the presence of accessory foramina, and the IOF location with respect to anatomic landmarks in order to guide surgeons, especially those operating on the Lebanese population during various procedures, and minimize complications.

## Materials and methods

This cross‑sectional retrospective study was conducted on cone-beam computed tomography (CBCT) scans of 105 Lebanese adult patients attending a private specialized imaging center in Beirut, Lebanon. Patients were referred from different clinics for radiological diagnosis, with a range of diverse indications (implants, impacted wisdom teeth, planning of orthodontics, and so on). All the patients were informed that the radiographs might be anonymously used for scientific purposes at a later stage and their consent was obtained. Moreover, due to the retrospective nature of this study, it was granted an exemption in writing by the ethical committee of the specialized imaging center in Beirut.

The inclusion criteria included:

(a) age: 18 years or older;

(b) absence of any history of trauma in the head and neck region;

(c) absence of congenital anomalies or syndromes with signs in the head and neck region;

(d) and patients who have no pathologic formation in the relevant region.

(e) nationality: Lebanese

One-hundred five CBCT images of 39 males and 66 females (a total of 208 IOF), with ages ranging from 18 to 72 years, met the inclusion criteria and were included in the study.

After scanning, the images were assessed in the axial, sagittal, and frontal planes to make sure that the anatomical structure is the IOF.

Many parameters were evaluated in the analysis of the IOF, including:

- The shape of the infraorbital foramen and its direction with respect to the midline.

**Figure 1:**
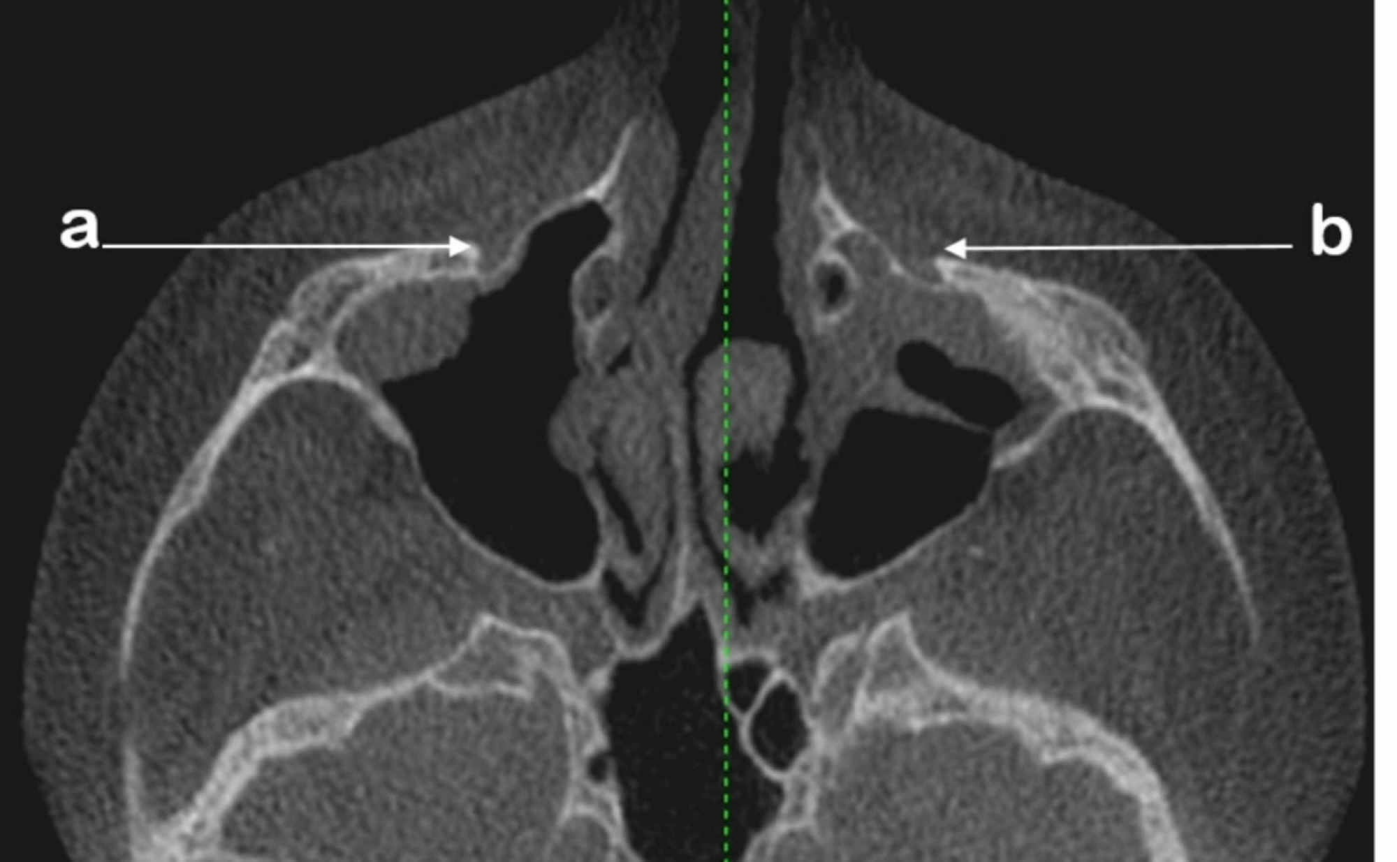
An axial cut showing the infraorbital foramen and its direction with respect to the midline a: oval vertical b: oval oblique

Foramina and their images in the coronal section were assessed and classified as ovals or circles. Those in a semi-circular shape were included in the circle class. In addition, IOFs with an oval shape were classified as oblique, vertical, and horizontal after studying their direction with respect to the midline (Figure 1).

**Figure 2:**
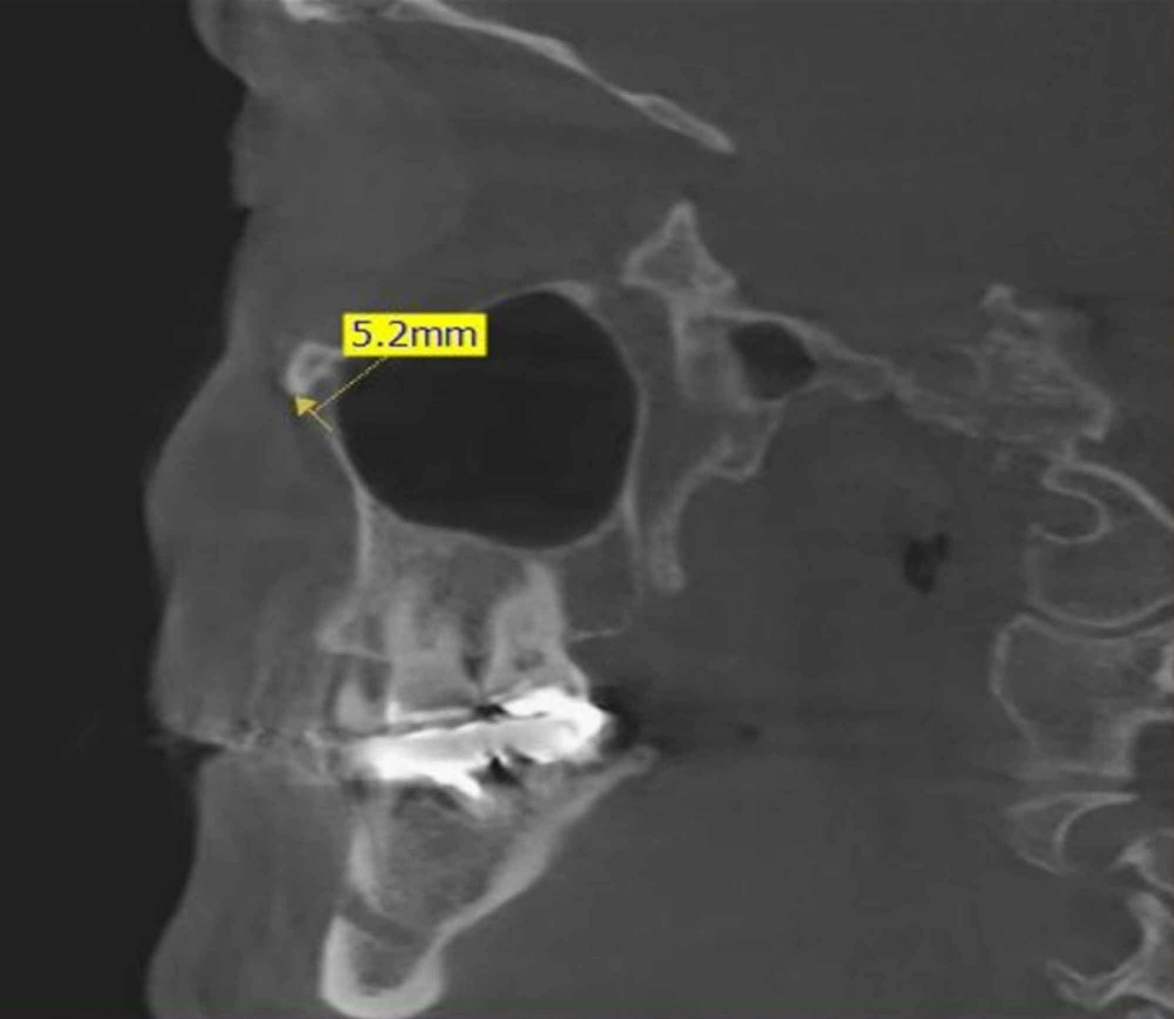
A sagittal view showing the diameter of infraorbital foramen by measuring the distance at the opening of the foramen

**Figure 3:**
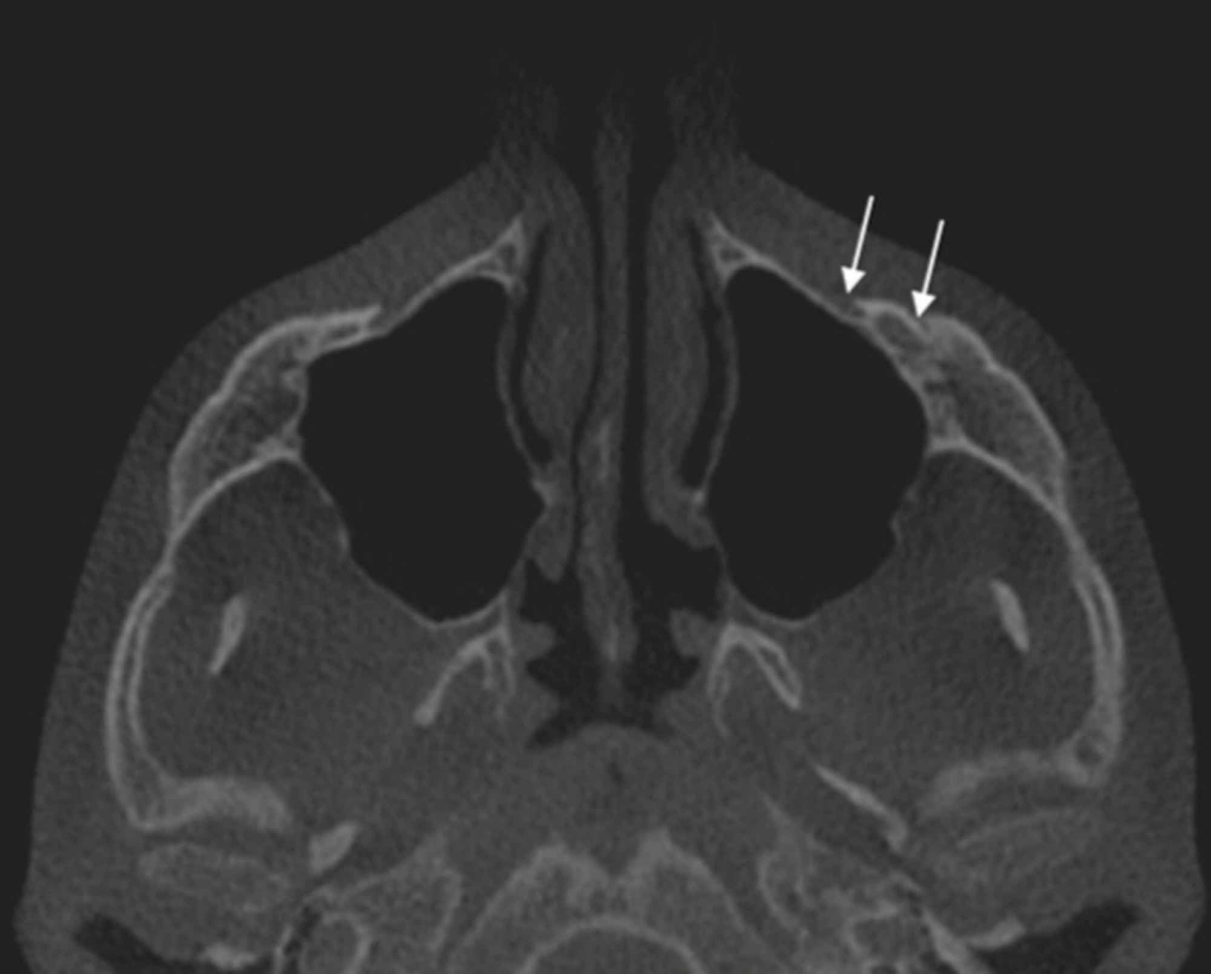
Axial cut showing two accessory foramina

- Opening diameter: The anteroposterior diameter of the IOF was measured in the sagittal view by measuring the distance at the opening of the foramen (Figure 2).

- Accessory foramen: The incidence of accessory IOF was studied on axial sections (Figure 3).

- Distances of the infraorbital foramen to several anatomic points: measurements were done on the coronal sections:

· Distance to the midline: The distance between the midline, where the two maxillary bones join, and the IOF was measured.

· Distance to the infraorbital edge (margin) (IOM): the distance between the IOF and the lower edge of the orbit was measured.

**Figure 4:**
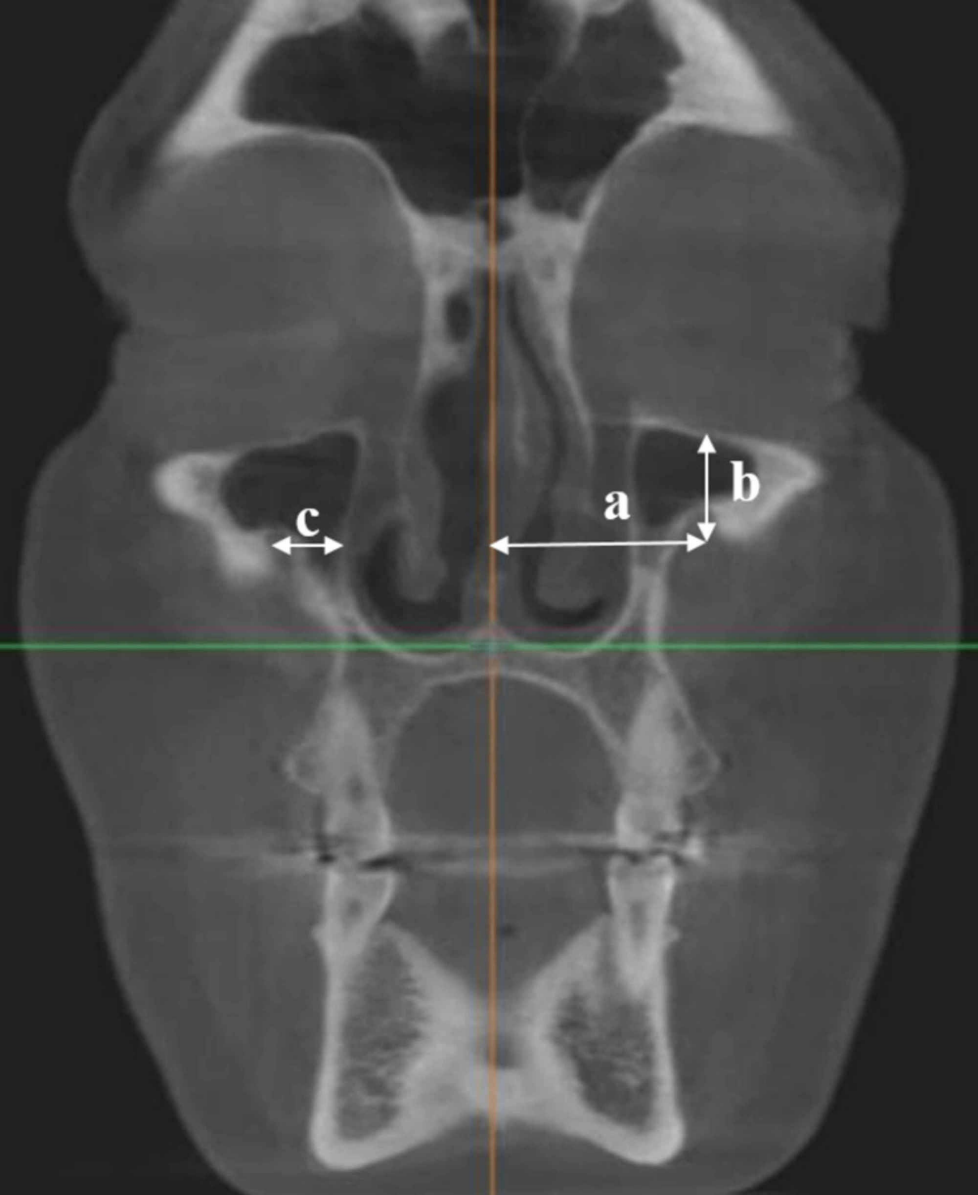
The distances of infraorbital foramen to several anatomic points a: to midline b: to infraorbital margin c: to lateral nasal wall.

· Distance to the lateral nasal wall (LNW): the distance between the IOF and the lateral nasal wall was measured (Figure 4).

To ensure the diagnostic reproducibility of the intraobserver reliability of the investigator, a total of 21 randomly chosen CBCTs (20%) were selected to assess measurement errors, and all measurements were repeated by the examiner two weeks after the first readings. Intra-examiner reproducibility was found to be 100%.

One investigator carried out all measurements on the right and left IOF in each CBCT image. He is a maxillofacial radiologist with more than 15 years of experience.

Statistical analysis

In this study, SPSS version 21 (IBM Corp., Armonk, NY, US) software was used. Results were expressed as mean and standard deviations (SD). The Shapiro-Wilk normality test was used to assess whether the quantitative variables follow a normal distribution (p > 0.05). The independent t-test was used to study the differences between gender and side while the one-way variance analysis test was used to study age groups. Finally, the Pearson chi-squared test was used to assess the shape of the IOF with respect to gender and side and to study the accessory foramen in terms of gender.

The value is considered statistically significant if p < 0.05.

## Results

In our study, the CBCT images of 105 patients, whose ages varied between 18 and 72 years old (mean age: 42.1), were examined.

Concerning the distances from the IOF to the anatomic landmarks, the distance from the IOF to the infraorbital margin measured between 3.4 mm and 11.2 mm, with a mean of 7.98 ± 1.41 mm. The distance between the IOF and the lateral nasal wall ranged between 4.99 mm and 18.20 mm, with a mean of 10.61 ± 2.39 mm, and, finally, the distance between the IOF and the middle of the face ranged between 19.60 mm and 32.40 mm, with a mean of 24.71 ± 2.09 mm.

When these distances were compared, no statistical difference was present between the right and left sides in all age groups (Table 1 and Table 3). When distances were compared with respect to genders, only a statistical difference was identified in the distance between the IOF and the lateral nasal wall (p=0.00), and the distance between the IOF and the middle of the face (p=0.016) (Table 2). For the shape of the IOF, 54.8% of the IOF were circular in shape, 28.6% were ovular in shape, and in the oblique direction, 13.3% were ovular in shape and in the vertical direction, and 3.3% were also ovular in shape but in the horizontal direction. The distribution of the shape showed a statistically significant difference with respect to gender and side (Tables 1-2). The circular shape is the most common shape in females, whereas the circular and oval oblique shapes have equal percentages in males. However, a similar distribution was observed with respect to different age groups (Table 3).

**Table 1:** Different morphometric variables according to the maxillary side IOF: Infraorbital foramen, IOB: Infraorbital border, NW; Nasal wall, MOF: Middle of face

Variable	Right	Left	P-value
Distance IOB to IOF (mm)	7.94 ± 1.45 (3.40-10.80)	8.03 ± 1.37 ( 4.50-11.20)	0.64
Distance IOF-NW (mm)	10.64 ± 2.22 (5.40-18.20)	10.58 ± 2.55 (4.90-18.00)	0.86
Distance IOF-MOF (mm)	24.94 ± 1.86 (20.80-29.70)	24.48 ± 2.28 (19.60-32.40)	0.11
Shape (%)	Circular: 57.1	Circular: 52.4	0.00
	Ovalvertical: 13.3	Ovalvertical: 13.3	
	Ovaloblique: 27.6	Ovaloblique: 29.5	
	Ovalhorizontal: 1.9	Ovalhorizontal: 4.8	
Accessory foramen 0 (%)	93.3	0: 89.5	0.04
1	6.7	8.6	
2	0	1.9	

**Table 2:** Different morphometric variables according to gender IOF: Infraorbital foramen, IOB: Infraorbital border, NW; Nasal wall, MOF: Middle of face

Variable	Male	Female	P-value
Distance IOB to IOF (mm)	7.94 ± 1.31	8.01 ± 1.47	0.75
Distance IOF-NW (mm)	11.63 ± 2.40	10.02 ± 2.17	0.00
Distance IOF-MOF (mm)	25.15 ± 2.08	24.43 ± 2.06	0.016
Shape (%)	Circular: 35.9	Circular: 62.1	0.01
	Oval vertical: 23.1	Oval vertical: 7.6	
	Oval oblique: 35.9	Oval oblique: 25.8	
	Oval horizontal: 5.1	Oval horizontal: 4.5	
Accessory foramen (%) No	82.1	93.9	0.09
Yes	17.9	6.1	
Diameter right (mm)	3.87±0.57	3.62±0.60	0.04
Left	3.87±0.70	3.62±0.64	0.06

**Table 3:** Different morphometric variables according to age groups IOF: Infraorbital foramen, IOB: Infraorbital border, NW; Nasal wall, MOF: Middle of face, C: Circular, V: Oval vertical, O: Oval oblique, H: Oval Horizontal

			<20	20-29	30-39	40-49	50-59	>60
Distance IOB-IOF (mm)	R		7.93±1.50	8.10±1.62	7.97±1.21	8.32±1.48	7.34±0.85	7.85±1.77
	L		8.01±1.65	8.35±1.23	8.18±1.38	8.07±1.27	7.80±1.24	7.69±1.54
Distance IOF-MOF (mm)	R		24.29±1.81	24.45±1.87	25.85±2.00	25.11±1.51	25.14±1.82	25.36±2.14
	L		23.64±2.09	24.19±2.50	24.21±1.93	24.36±2.12	25.14±2.29	25.62±2.38
Distance IOF-LNW (mm)	R		10.21±1.72	10.82±2.25	11.21±2.97	10.39±1.88	11.10±2.71	10.34±2.13
	L		10.30±2.43	10.55±2.40	10.99±2.78	10.51±2.73	11.11±2.72	10.20±2.60
Diameter (mm)	R		3.76±0.58	3.52±0.61	3.41±0.49	4.00±0.58	3.73±0.61	3.75±0.60
	L		3.69±0.67	3.81±0.60	3.33±0.65	3.85±0.65	3.54±0.78	3.89±0.61
Shape (%)	R	C	63.2	50	36.4	57.1	64.7	66.7
		V	15.8	13.6	18.2	9.5	5.9	20
		O	21.1	36.4	45.5	28.6	29.4	6.7
		H	0	0	0	4.8	0	6.7
	L	C	68.4	54.5	54.4	23.8	64.7	53.3
		V	10.5	9.1	18.2	23.8	17.6	0
		O	15.8	36.4	27.3	42.9	11,8	40
		H	5.3	0	0	9.5	5.9	20

In relation to the accessory foramen, it is present in 8.6% of the studied foramina. Seven point six percent of the foramina has one accessory foramen while 1% have two accessory foramina. The accessory foramina showed a similar distribution with respect to gender (Table 2) but a statistically significant difference was present with respect to the maxillary side (Table 1).

Finally, the diameter of the foramina was measured in the sagittal plane. The mean diameter was 3.71 ± 0.63 mm and this diameter did not show any statistically significant difference in all age groups (Table 3).

## Discussion

The IOF is an important structure in the maxillofacial area where the infraorbital artery and nerve pass. These structures supply various areas in the face [12]. The foramina demonstrate morphologic variations between different populations [13]. Thus, it is essential for surgeons and anesthesiologists to have exact knowledge of the location of the IOF before any procedure [12]. Various anatomic landmarks are used in several studies to identify its location and help surgeons in reconstructive and orthognathic surgeries, as well as while performing a nerve block [11].

Multiple studies discussed the location of the IOF with respect to the infraorbital border. The mean distance varied between 5.7 mm and 10.9 mm in different populations [14]. In a study performed on 242 crania in Brazil, this distance measured 8 mm and showed no variability in terms of gender or side [12].

Similarly, the mean distance measured 8.8 mm in a study performed on 20 dry skulls in Turkey [15]. However, mean distances as small as 5.7 mm and 6.7 mm were reported in other studies [16-17].

On the other hand, multiple studies showed no statistical differences between sides, and mean values of 6.28 ± 1.79 mm and 6.45 ± 1.76 mm were measured by Macedo et al. on the right and left sides of the maxilla, respectively [18]. However, a significant difference was present in a study done by Bahsi et al. between the left and right sides of the maxilla. The distance on the left measured 7.39 mm, whereas this value was 7.47 mm on the right [14]. In another study done by Dagistan et al., the mean distance was found to be 5.64 ± 1.78 mm, without a significant difference between the right and left sides and between genders [19].

In our study, the mean distance between the IOF and infraorbital border showed some variability with respect to side (Table 1), gender (Table 2), and age groups (Table 3); however, no statistical difference was present in any subgroup.

Concerning the distance of the IOF to the lateral nasal wall, the mean distance measured 9.70 ± 2.76 mm on the right side and 8.95 ± 2.54 mm on the left side. In this previous study by Dagistan et al. done on CBCT images, no statistical difference was found between genders, but a statistical difference was found between the left and right sides [19]. In our study, this distance measured 10.64 ± 2.22 mm on the right side and 10.58 ± 2.55 mm on the left side. Statistical analysis showed a similar distribution on both sides. However, a statistical difference was present with respect to gender (p=0.00) (Table 2). This is in contrast to the preceding study done on the Turkish population where no statistical difference was present between genders [19]. Therefore, a larger distance is to be estimated in men. This is similar to a study done on human skulls where all mean distances were larger in males [20]. Another study done by Lee et al. showed that the IOF location varied significantly among genders and changed significantly within the first years of life. However, this change leveled off after the age of 20 years [21]. In our study, the IOF location did not change significantly with age, and all age groups demonstrated a similar distribution in all measured distances. It is worth noting that our entire sample was composed of patients older than 18 years.

Different values measuring the distance between the middle of the face and the IOF are present. This may be due to the anatomical differences between different ethnicities [12]. This distance was found to be 25.69 ± 2.37 in a study done by Aggarwal et al. [5]. Larger values were reported by Gupta, where the mean distance was 28.5 ± 2.6 mm [22]. Statistical difference was present in some studies between genders, and others showed a statistical difference between sides [20,23]. There was no significant difference between the sides and genders in the study conducted by Dagistan et al., where the mean distance was 25.10 ± 2.17 mm [19]. In our study, this distance was not considered statistically significant with respect to the maxillary side (Table 1). However, the measured distance was statistically significant with respect to genders (Table 2). We can see that men had greater distances between the IOF and the middle of the face, just like the distance between the IOF and the lateral nasal wall.

The frequency of the existence of accessory IOF varied in the literature between 0.8% and 27.3% [8]. This value should be remembered by all surgeons and anesthesiologists during maxillofacial surgeries and while performing a nerve block [8]. A single accessory foramen was observed in 11.5% of the foramina, and double foramina were observed in 1.28% of the foramina in a Turkish specimen [24]. In a Brazilian study on 242 dry skulls, accessory foramina were present in 10.7% of the specimens without any significant difference with respect to gender or the side of the maxilla [12]. Another study reported the presence of an accessory foramen more frequently on the right [25]. In our study, 91.4% of the foramina were not associated with an accessory foramen. In 7.6% of the cases, one accessory foramen was present, and in 1% of the cases, two accessory foramina were present. These accessory foramina were equally distributed between the genders but occurred more frequently on the left side of the maxilla where one accessory foramen was present in 8.6% of the cases and two foramina in 1.9% of the cases.

The shape of the IOF also shows some variability. The foramina presented an oval shape in 50% of the cases, a semicircle in 29.2% of the cases, and a circular shape in 20.8% of the cases in a study done by Apinhasmit et al. [26].

Another study showed that the oval shape is the most common one, where the oval vertical shape represented 42.7% of the cases, the oval horizontal shape represented 28.1% of the cases, and, finally, the circular shape was present in 29% of the cases [27]. Dagistan et al. also showed that the oval shape is the most common shape present in 58% of the cases [19]. Similarly, the oval shape was the most common shape in a Sri Lankan study [11]. In our study, however, the circular shape was the most common shape present in 54.8% of the cases. The only statistically significant difference was found with respect to gender and side. The circular shape was the most common shape in females of the Lebanese population (p=0.01), at 39%. With regard to sides, the circular shape was also more common on the right side (p=0.00).

Finally, the transverse diameter of the IOF measured 2.87 ± 0.78 mm and 3.71 ± 0.61 mm in different studies [19,28]. In our population, the mean transverse diameter measured 3.71 ± 0.63 mm, and the values ranged between 2.26 mm and 5.44 mm. The mean value was comparable to a study done on the Turkish population [19]. The transverse diameter did not show a significant difference with respect to different age groups (p=0.07 on the right side and p=0.2 on the left side). However, a statistically significant difference was present with respect to gender only on the right side of the maxilla (p=0.04), where a larger diameter was present in males. However, Kara et al. noted statistically significant differences between genders where larger mean values were found in males in both maxillary sides [29]. On the other side, Nanayakkara et al. reported a mean diameter of 3.06 ± 0.72 in males and a value of 3.17 ± 0.51 in females. These values were not considered statistically significant and no difference was noted between genders nor between maxillary sides [11].

However, our study possesses some limitations. Our study reviewed the CBCT images from a single referral center in Beirut and the sample was a small one with the need for a larger sample in future studies to define the characteristics of the IOF in the Lebanese population. Moreover, the sample contained a larger number of females as compared to males.

## Conclusions

The IOF is an anatomical foramen in the head and neck region. The infraorbital nerve and artery pass through it. This foramen shows a lot of variability between different populations. These differences in the morphometric characteristics of the IOF may be due to racial differences and the variability of the methods used in each study. Most studies available in the literature are conducted on dry skulls and on cadavers. Images possessing lower section thickness and lower section intervals with higher resolution can be acquired through the CBCT technique, which ensures a more detailed examination. Therefore, it gives successful results in identifying the anatomic characteristics of the IOF. Other advantages of CBCT include emitting less radiation, having less cost, and being easier to use as compared to CT. To the best of our knowledge, no studies are present in the Lebanese population when the differences between populations are considered. Our study reveals the anatomic characteristics of the IOF in a sample of the Lebanese population by utilizing the advantages of CBCT and supports the fact of variation of the location of the IOF. Thus, the exact location should always be remembered during an ION block, during maxillofacial surgeries, and during esthetic procedures involving the facial region, in order to prevent unnecessary complications.
